# The first single-incision plus one-port transverse colon resection using Senhance Digital Laparoscopy System: a case report

**DOI:** 10.1186/s40792-021-01167-x

**Published:** 2021-04-13

**Authors:** Yume Minagawa, Yasumitsu Hirano, Atsuko Kataoka, Satoshi Shimamura, Masahiro Kataoka, Masahiro Asari, Takatsugu Fujii, Shintaro Ishikawa, Toshimasa Ishii, Hiroshi Sato, Shinichi Sakuramoto, Kojun Okamoto, Isamu Koyama

**Affiliations:** Department of Gastroenterological Surgery, Saitama Medical University International Medical Center, 1397-1 Yamane, Hidaka-shi, Saitama, 350-1298 Japan

**Keywords:** Senhance system, Colorectal cancer, Single-incision laparoscopic surgery

## Abstract

**Background:**

We have introduced the Senhance Digital Laparoscopy System and actively use for colorectal cancer surgery. Recently, we also try to perform surgery by reduce port as less invasive method. For the first time, we report a case of single-incision plus one-port transverse colectomy using Senhance system.

**Case presentation:**

The case was a 57-year-old woman, diagnosed with transverse colon cancer referred to our department. The preoperative diagnosis was cT1bN0M0, Stage I. We performed single-incision plus one-port transverse colon resection using Senhance system without any problems. The operative time was 203 min and the blood loss was 35 ml.

**Conclusion:**

We report the first case of single-incision plus one-port transverse colectomy using Senhance system. We trust this approach will find increasing use, enabling a safer means of minimally invasive robotic surgery.

## Background

The Senhance Digital Laparoscopy System (TransEnterix Surgical Inc, Morrisville, NC, USA) introduced at our hospital in Japan is a new surgical support robot and an alternative to the da Vinci Surgical System (Intuitive Surgical Inc, Sunnyvale, CA, USA). It has been approved by the Japanese Ministry of Health, Labor & Welfare (May 2019), earning insurance coverage in July 2019 for 98 types of laparoscopic surgery.

This system was developed as an extension of the laparoscope to ensure greater procedural stability (without tremor) and reap benefits other than economic efficiency. The latter includes a capacity to transmit loads (i.e., tension and pressure) applied to forceps tip or handle (tactile feedback system), which the da Vinci system lacks, and an eye-tracking camera by which surgeons freely control the endoscope visually. Neck and back strain during console maneuvers are thus reduced.

Although waning in popularity, the superior aesthetic outcomes of reduced-port surgery (i.e., single-incision laparoscopic surgery) have fueled its broad usage in colorectal cancer surgery since ~ 2010. We have performed single-incision laparoscopic colorectal surgery on more than 1000 patients and documented the utility. In addition to improved aesthetics and the distinct advantages of minimal invasion, such procedures are also solo events that do not require added assistance.

Various European and US institutions have integrated the Senhance system in colorectal cancer surgery while still relying on assistants. Because digitalization is the thrust of this system, we believe a technique that eliminates analog input by an assistant is clearly warranted in this setting, and we have been actively working towards that end. Adding to our prior publication on single-incision plus two-port surgery for sigmoid colon cancer, we now offer the first account of transverse colon cancer resection using a single incision and a single port.

## Case presentation

The patient was a 57-year-old woman (height, 155 cm; weight, 36.1 kg; body mass index, 15.0) with biopsy-proven adenocarcinoma of the transverse colon, referred to our department for treatment. She was not in distress (performance status, 0; American Society of Anesthesiologists score, 1). Her past medical history included psoriasis and urolithiasis. Family history, lifestyle, and medication use were non-contributory.

Preoperative blood tests revealed the following: white blood cells (WBC), 6720 cells/μl; hemoglobin, 13.9 g/dl; platelet count, 212 × 10^3^ cells/μl; C-reactive protein, 0.01 mg/dl; albumin, 4.2 g/dl; carcinoembryonic antigen (CEA), 2.5 ng/ml; and Carbohydrate antigen19-9(CA19-9), 18.7 U/ml. Tests assessing physiologic functions were not abnormal. Lower gastrointestinal endoscopy exposed a 30-mm lesion of transverse colon that likely invaded submucosa (Paris Classification, 0-Is + IIc). Computed tomography (CT) imaging also disclosed mural thickening and contrast effect at mid-transverse colon, but there were no signs of metastasis. Based on the Japanese Classification of Colorectal, Appendiceal, and Anal Carcinoma (9th edition), the preoperative diagnosis was Stage I (cT1b, N0, M0) cancer of transverse colon. We then chose the Senhance system for transverse colon resection.

## Surgical procedure

**Fig. 1:**
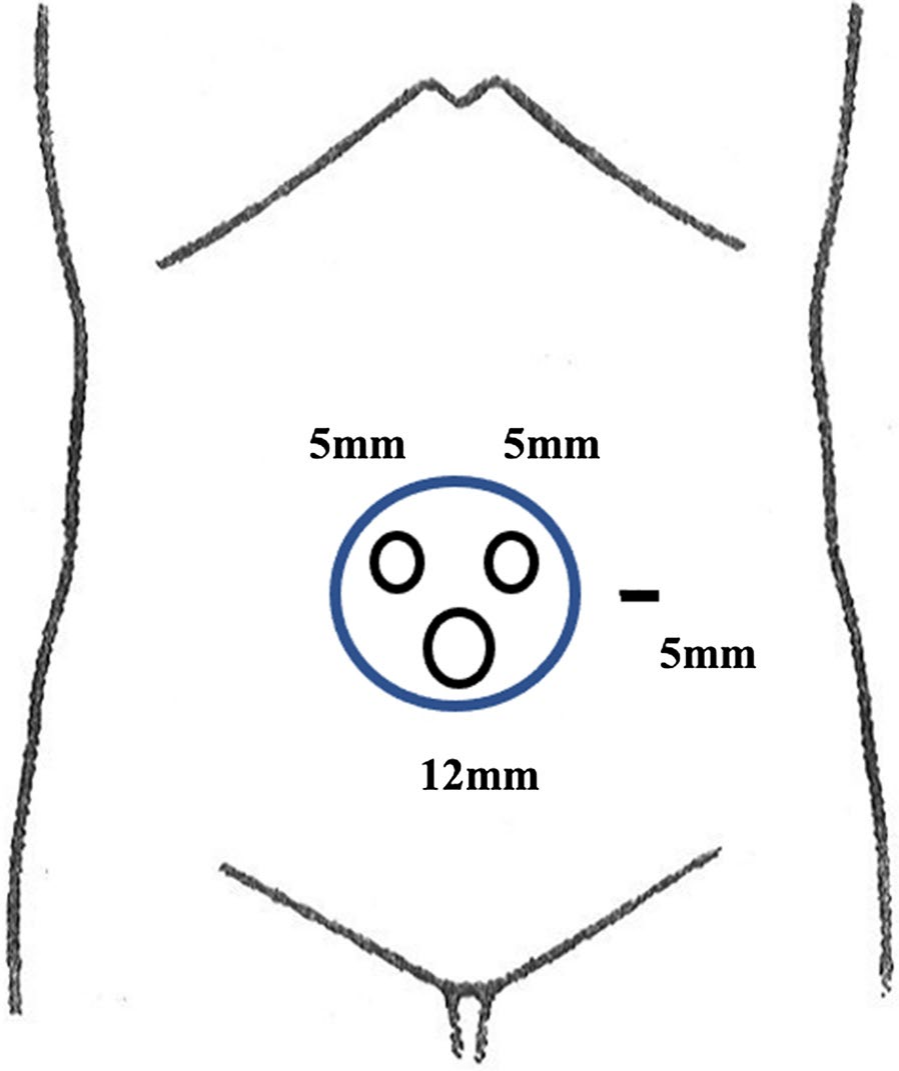
Port placement. A 12-mm camera port and two 5-mm ports were introduced by Free Access device at umbilicus and a 5-mm port was inserted at left side of abdomen

**Fig. 2:**
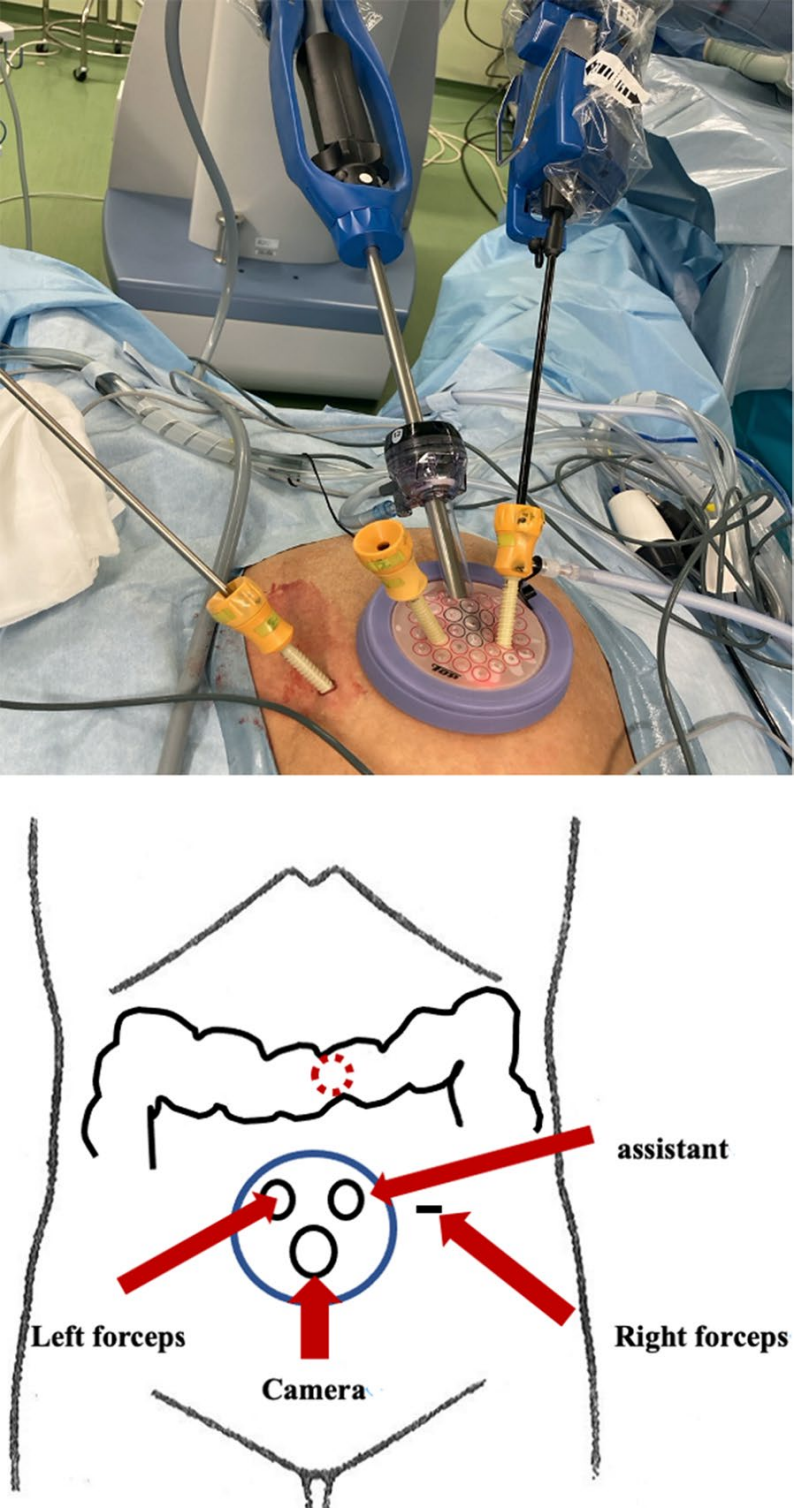
Positional relationship between forceps and lesions. Tumor location was verified as mid-transverse colon. The camera and forceps were inserted as shown in the above figure

The patient was placed in lithotomy position under general anesthesia for a mini-laparotomy, performed via 3-cm longitudinal incision at umbilicus. A 12-mm camera port and two 5-mm ports were introduced by Free Access device (TOP Corp, Tokyo, Japan), and a 5-mm port was inserted at left side of abdomen, thus constituting a single-incision plus one-port procedure (Figs. 1, 2). Tumor location was verified as mid-transverse colon. The abdominal cavity was otherwise free of overt metastasis or ascites.

**Fig. 3:**
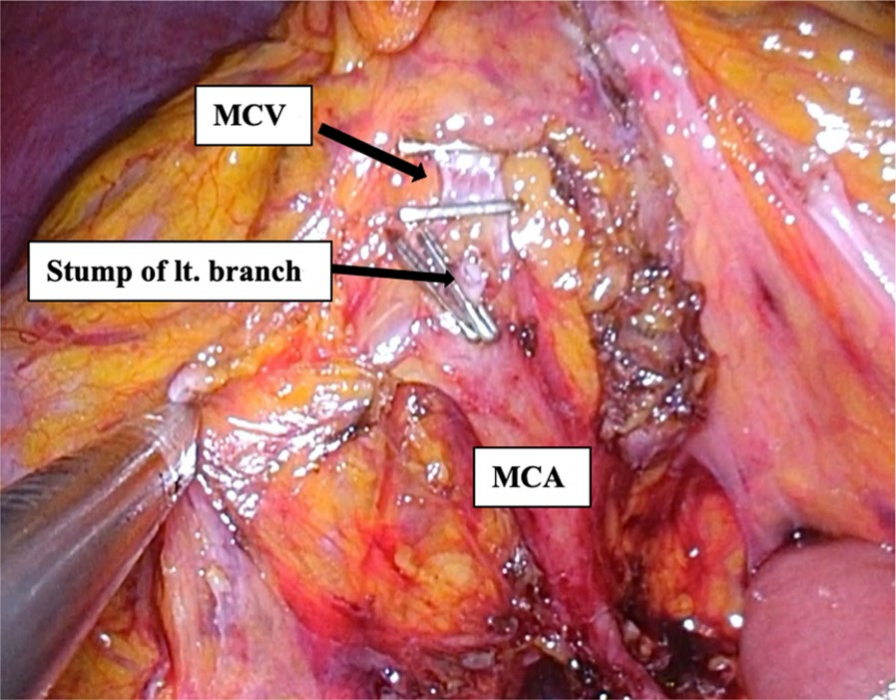
Intraoperative findings. The left arterial branch of MCA was dissected at bifurcation and MCV was dissected at the same level after the lymph node dissection

The patient was then placed in head-down, left-oblique position to shift the small bowel away and secure the view. Under robotic aid, duodenum and middle colic artery (MCA) were identified, and the mesentery was detached from duodenal/pancreatic moorings. After exposing the root of middle colic artery, D3 lymph node dissection took place, including all adipose tissue to the point of bifurcation on the side of resection (Fig. 3). The left arterial branch was then clipped (at bifurcation) and dissected, thereafter identifying and dissecting middle colic vein (MCV, dorsal aspect of artery) at the same level. Finally, the lesser sac was opened and the transverse colon fully mobilized.

Having completed the robotic operative phase, we reverted to extracorporeal control. The transverse colonic mesentery was removed proximal and distal to the lesion (10 cm each way), and the colon was dissected for tumor resection. We then performed a functional end-to-end anastomosis (FEEA), leaving the mesentery open. Once hemostasis was achieved, the wound was closed to complete the operation. Operative time was 203 min, docking time was 11 min, duration of console operation was 93 min, and blood loss was 35 ml.

On the second postoperative day, emergency lower gastrointestinal endoscopy was initiated for anastomotic bleeding. We found no obvious source, suggesting spontaneous resolution. The patient resumed eating on postoperative day 5 and was discharged on postoperative day 8. The final histopathologic diagnosis was Stage IIIa (pT1b, N1a [1/19], M0) carcinoma of transverse colon, margins of resection clear.

## Discussion

Recently introduced robotic surgical systems, especially the da Vinci system, have flourished and gained worldwide acceptance. In robot-assisted surgery, tremor reduction and motion scaling functions allow greater surgical precision. Furthermore, surgeons may adjust the field of view while perusing high-definition 3D images, enabling more stress-free and accurate surgical performance.

Our hospital was the first in Japan to introduce the Senhance Digital Laparoscopy System, having performed colorectal resections in roughly 40 patients. Aside from aforementioned benefits of robot-assisted surgery, another major feature is the capacity for tactile feedback [1]. In Europe and the US, the Senhance system already has been availed for various surgeries, including urologic and gynecologic operations [2]; and its safe implementation in gastrointestinal surgery is reported as well [3]. However, there are few accounts of colorectal resections in Japan.

As with other colorectal resections involving Senhance system support [4], colorectal resections at our hospital have mostly been four-port operations [5, 6]. Nevertheless, our past experience with single-incision laparoscopic colorectal cancer surgery and our pursuit of even less intrusion through digitalization (integral to this system) have fostered the concept of reduced-port surgery, with no required surgical assistance (since December 2019). In June 2020, we reported an instance of single-incision plus two-port robotic surgery for cancer of the sigmoid colon [7].

Herein, the first documented use of single-incision plus one-port transverse colon resection is detailed. Given this restricted access (i.e., left-sided abdominal port, Free Access device), we safely and accurately conducted a fairly difficult transverse colonic arterial dissection using robotic forceps. The 5-mm port of Free Access sufficed for any needed assistance, eliminating a separate port for this purpose. Our approach prevents inadvertent organ damage by an assistant's forceps or untoward interference during forceps manipulation, not only improving procedural safety but also reducing operator stress.

In an ongoing clinical trial of da Vinci colectomy in Japan, anastomosis is performed intra-corporeally. However, the length of the umbilicus incision in the present case was 3 cm, which was considerably shorter than the length of the umbilicus incision reported by conventional laparoscopic colectomy [8], so an intra-corporeal anastomosis was not performed to shorten the incision. In addition, if the umbilicus incision is shorter, the forceps and scope will interfere with each other, making it difficult to further shorten the wound with this reduced port procedure with Senhance.

This system has limitations in terms of robotic-arm range of motion and forceps tip configuration, which at present cannot be bent. Such drawbacks may be problematic for some tumor locations and patient physiques. However, we hope to pursue minimally invasive techniques for more procedures going forward, working to improve this system and harnessing its unique advantages.

## Conclusion

We present the first single-incision plus one-port transverse colon resection as primary cancer treatment with Senhance system. We trust this approach will find increasing use, enabling a safer means of minimally invasive robotic surgery.

## Data Availability

Data sharing is not applicable to this article, since data sets were neither generated nor analyzed for the case series.

## Abbreviations

WBC: White blood cells
CEA: Carcinoembryonic antigen
CA19-9: Carbohydrate antigen19-9
CT: Computed tomography
MCA: Middle colic artery
MCV: Middle colic vein
FEEA: Functional end-to-end anastomosis
3D: Three dimensional
